# Intravitreal Injection of Long-Acting Pegylated Granulocyte Colony-Stimulating Factor Provides Neuroprotective Effects via Antioxidant Response in a Rat Model of Traumatic Optic Neuropathy

**DOI:** 10.3390/antiox10121934

**Published:** 2021-12-01

**Authors:** Chin-Te Huang, Yao-Tseng Wen, Tushar Dnyaneshwar Desai, Rong-Kung Tsai

**Affiliations:** 1 Institute of Medical Sciences, Tzu Chi University, Hualien 970, Taiwan; cshy1795@csh.org.tw; 2 Institute of Eye Research, Hualien Tzu Chi Hospital, Buddhist Tzu Chi Medical Foundation, Hualien 970, Taiwan; ytw193@tzuchi.com.tw (Y.-T.W.); tushardesai.desai@gmail.com (T.D.D.); 3 Department of Ophthalmology, Chung Shan Medical University Hospital, School of Medicine, Chung Shan Medical University, Taichung 402, Taiwan; 4 Doctoral Degree Program in Translational Medicine, Tzu Chi University and Academia Sinica, Hualien 970, Taiwan

**Keywords:** traumatic optic neuropathy, oxidative stress, neuroinflammation, neurodegeneration, pegylated granulocyte colony-stimulating factor, microglia, anti-inflammation, anti-apoptosis, anti-oxidation

## Abstract

Traumatic optic neuropathy (TON) may cause severe visual loss following direct or indirect head trauma which may result in optic nerve injuries and therefore contribute to the subsequent loss of retinal ganglion cells by inflammatory mediators and reactive oxygen species (ROS). Granulocyte colony-stimulating factor (G-CSF) provides the anti-inflammatory and anti-oxidative actions but has a short half-life and also induces leukocytosis upon typical systemic administration. The purpose of the present study was to investigate the relationship between the anti-oxidative response and neuroprotective effects of long-acting pegylated human G-CSF (PEG-G-CSF) in a rat model of optic nerve crush (ONC). Adult male Wistar rats (150–180 g) were chosen to have a sham operation in one eye and have ONC in the other. PEG-G-CSF or phosphate-buffered saline (PBS control) was immediately administered after ONC by intravitreal injection (IVI). We found the IVI of PEG-G-CSF does not induce systemic leukocytosis, but increases survival of RGCs and preserves the visual function after ONC. TUNEL assays showed fewer apoptotic cells in the retina in the PEG-G-CSF-treated eyes. The number of sorely ED1-positive cells was attenuated at the lesion site in the PEG-G-CSF-treated eyes. Immunoblotting showed up-regulation of p-Akt1, Nrf2, Sirt3, and HO-1 in the ON of the PEG-G-CSF-treated eyes. Our results demonstrated that one IVI of long-acting PEG-G-CSF is neuroprotective in the rONC. PEG-G-CSF activates the p-Akt1/Nrf2/Sirt3 and the p-Akt1/Nrf2/HO-1 axes to provide the antioxidative action and further attenuated RGC apoptosis and neuroinflammation. This provides crucial preclinical information for the development of alternative therapy with IVI of PEG-G-CSF in TON.

## 1. Introduction

Traumatic optic neuropathy (TON) refers to any insult to the optic nerve secondary to direct or indirect head or ocular injury [1]. TON is an uncommon cause of visual loss with a reported incidence of 0.7% to 2.5% [2,3,4]. There is no consensus among ophthalmologists regarding the standard treatments of TON [5,6], with regards to steroids [1,7] or surgery [8].

The reactive oxygen species (ROS) produced mainly by mitochondria are required to maintain normal physiological functions, including gene expression, signal transduction, cell proliferation, and host defense [9]. Excessive ROS production and oxidative stress can induce cell apoptosis by damaging mitochondrial DNA, inhibiting the mitochondrial respiratory chain transition, and increasing mitochondrial membrane permeability [10]. This is involved in the pathogenesis of many neurodegenerative diseases such as Alzheimer’s disease [11], Parkinson’s disease [12], and many kinds of eye diseases [13]. Since TON is usually associated with traumatic brain injury (TBI), a mini-review proposed that there are some connections of endoplasmic reticulum (ER) and oxidative stress to retinal degeneration, TBI, and TON [14]. A sonication-induced TON model has linked an increase in ROS and oxidative stress to retinal and optic nerve degeneration [15]. Ahmad et al. also confirmed the increase of oxidative stress in mice retina with TON [16]. Therefore, ROS may play a role in the TON pathogenesis [13].

We previously demonstrated that systemic administration of granulocyte colony-stimulating factor (G-CSF), an old drug used to recruit the hematopoietic progenitors from bone marrow to peripheral blood, has neuroprotective effects not only in a rat model of anterior ischemic optic neuropathy (rAION) [17,18,19,20] but also in an optic nerve crush (ONC) model [21,22,23]. Our previous studies have shown that systemic administration of G-CSF may contribute to anti-inflammation via decreasing inflammatory cell infiltration in the injured optic nerve and promoting anti-apoptosis of retinal ganglion cells (RGCs) via activation of PI3k/Akt signaling in the rat ONC model [22,23]. One of our studies also revealed that both G-CSF and G-CSF receptor (G-CSFR) are expressed in normal rat retina and exogenous G-CSF can enhance the anti-apoptotic effect on RGCs [21]. Apart from these protective effects, G-CSF is suggested to have an anti-oxidant effect through an increase in the interleukin 10 (IL-10) levels in vascular endothelial function [24,25]. G-CSF also demonstrates an important antioxidant effect in rats against adriamycin-induced cardiac, renal and hepatic toxicities by a decrease in the tissue malondialdehyde levels and an increase in the tissue glutathione levels [26]. Furthermore, G-CSF rescues the functional and morphologic deterioration of the neural retina in oxygen-induced retinopathy and attenuates oxidative stress-induced apoptosis in vascular endothelial cells [27]. However, the role of G-CSF against oxidative stress in the ONC model remains little known.

The systemic administration of human recombinant G-CSF needs repeated injections for its’ short circular half-life (3.5–3.8 h) [28], and systemic applications of G-CSF will induce leukocytosis [28]. Our rationale is that as pegylated G-CSF (PEG-G-CSF) has a longer half-life up to 42 h [28,29] and if it can be applied via an intravitreal injection (IVI) into the eye with the blood-ocular barrier, the occurrence of systemic side effects may decrease. However, does this work in the treatment of TON?

Our current study aimed to investigate the safety of IVI of PEG-G-CSF and its effect on prolonged neuroprotection. Furthermore, we wanted to examine the role of PEG-G-CSF in oxidative stress in the rat ONC model.

## 2. Materials and Methods

### 2.1. Animals

All animal care and surgical procedures were approved by the Institutional Animal Care and Use Committee at Tzu Chi Medical Center and managed in accordance with the Association for Research in Vision and Ophthalmology Statement for the Use of Animals in Ophthalmic and Vision Research. Male Wistar rats (BioLASCO Co., Taipei City, Taiwan) used in this study were around 150–180 g in weight. The rats were maintained under a controlled 12-h shift of the light-dark cycles and had free access to food and water in a controlled environment with a constant temperature of 23 °C and constant humidity of 55%. All operations were performed with the animals under general anesthesia, which was achieved by intramuscular administration of a mixture of ketamine (100 mg/kg body weight) and xylazine (10 mg/kg body weight; Sigma, St. Louis, MO, USA).

### 2.2. Study Design

**Figure 1:**
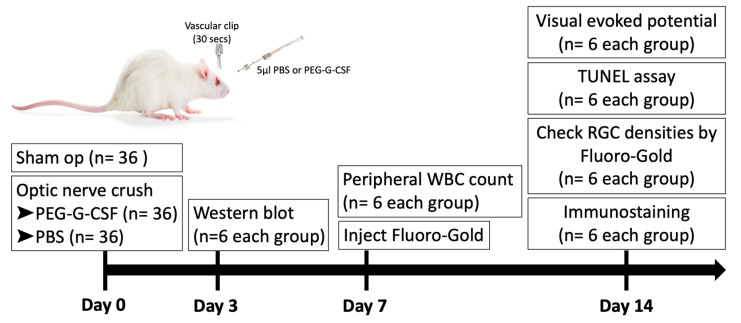
Brief summary of study design to investigate the role of intravitreal injection (IVI) of pegylated granulocyte colony-stimulating factor (PEG-G-CSF) in the rat optic nerve crush (ONC) model. PBS: phosphate-buffered saline. RGC: retinal ganglion cell. WBC: white blood cell.

A total of 108 rats (6/group/time point) were used in this study. 36 rats received sham operations. 72 rats were randomized into two equal groups after ONC in the right eye. The first group received IVI of 5 µL phosphate-buffered saline (PBS), and the second group received IVI of 5 µL PEG-G-CSF alone in the operated eyes one hour after ONC. All animals survived until the end of the experiment without developing any complications. Post-operation day 3, the optic nerve samples were collected for Western blot analysis. Post-operation day 7, peripheral blood samples were collected for white blood cell (WBC) counts. Post-operation day 14, visual function was assessed by performing flash visual-evoked potential (fVEP) analysis; the RGC density was measured by retrograde FluoroGold labeling. In situ TdT-dUTP nick end-labeling (TUNEL) assays in the RGC layer and immunohistochemistry of injured sites in the ON were also conducted. A brief summary of the study is illustrated in Figure 1.

### 2.3. Rat Optic Nerve Crush Model and Treatments

ONC injury was induced as described in our previous reports [7,21,22,30]. Briefly, the optic nerve was exposed, and a vascular clip (60-g microvascular clip, World Precision Instruments, FL, USA) was applied on the optic nerve posterior to the eyeball 2 mm for 30 s. The surgery was performed gently to avoid damage to the small paraoptic vessels. Post-operation, the rats were kept warm on the electric heating pads at 37 °C for recovery. The sham group of rats received optic nerve exposure without the crush.

Pegfilgrastim (Neulasta; Amgen Inc., Juncos Puerto Rico, Thousand Oaks, CA, USA) is a pegylated human colony-stimulating factor (PEG-G-CSF) produced by genetic recombination technology [29]. It works to selectively increase the amount of neutrophils, as well as increase their functional efficacy. Intravitreal injections were performed after rats were anesthetized. After pupil dilatation with topical 0.5% tropicamide and 0.5% phenylephrine hydrochloride eye drops (Mydrin-P; Santen Pharmaceutical Co., Ishikawa, Japan), a 33-gauge needle (Hamilton 7747-01 with a Gaslight syringe, IA2-1701RN 10 µL SYR; Hamilton Co., Hamilton, KS, USA) was passed through the sclera at the ora serrata level under the operating microscope. A total of 5 µL of PEG-G-CSF or phosphate-buffered saline (PBS) was injected directly into the vitreous cavity of each rat.

### 2.4. Flash Visually Evoked Potentials (fVEPs)

The procedure has been described in detail in our previously published reports [7,21,22,23,30,31]. Briefly speaking, after ONC, fVEPs were recorded 14 days later by a visual electrodiagnostic system (Espion, Diagnosys LLC, Gaithersburg, MA, USA). Background illumination was turned off with a flash intensity of 30 cd·s/m^2^, a single flash with a flash rate of 1.02 Hz, and a test average of 64 sweeps. The first positive wavelet was defined as the P1 component, and the first negative wavelet was defined as the N1 component. The peak-to-peak amplitudes of P1-N2 waves were measured and compared among the groups (n = 6 in each group) to evaluate visual function.

### 2.5. White Blood Cell Count of Peripheral Blood

Blood was drawn from cardiac puncture into the heparinized coated tubes by using a 23-gauge needle. The blood samples were collected for complete blood count (CBC) analysis on day 0 and day 7 later after different treatments. All manipulations were performed with animals under general anesthesia. Total WBCs were counted by the Cellometer K2 automated cell counter (Nexcelom Bioscience LLC, Lawrence, MA, USA) [21].

### 2.6. Retrograde Labeling of RGCs with Fluoro-Gold

The detailed steps have been described in our previous reports [7,21,22,23,30,31]. Briefly, the retrograde labeling of the RGCs was performed 7 days before the rats were euthanized on day 14. Injection of 2 μL of 5% Fluoro-Gold (Fluorochrome LLC, Denver, CO, USA) was delivered into each superior colliculus by using a Hamilton syringe. After the carbon dioxide euthanasia of rats, their eyeballs were harvested for retina preparation. The RGCs were measured at a distance of 1 mm from the center of the optic nerve head to provide the central RGC density. The numbers of RGCs in five randomly selected areas (62,500 μm^2^) in the central region of each retina were obtained to retrieve the mean RGC densities in the central retina (n = 6 in each group).

### 2.7. In Situ Terminal Deoxynucleotidyl Transferase dUTP Nick End Labeling (TUNEL) Assay for Apoptotic Cell Measurements

The TUNEL assays were performed under the protocol provided by the manufacturer (DeadEnd Fluorometric TUNEL System; Promega Corporation, Madison, WI, USA) to detect apoptotic cells. All the frozen retinal sections were collected at 1–2 mm anterior to the optic nerve head for further comparison. Ten high-powered fields (HPFs, ×400 magnification) were selected randomly to count the TUNEL-positive cells in the RGC layer of each sample. Then the average was calculated for further comparison.

### 2.8. Immunostaining at the Injury Site of Optic Nerves

The ectodysplasin 1 (ED1) antibody reacted against extrinsic macrophages [19,32,33], while the ionized calcium-binding adaptor molecule 1 (Iba1) was a marker of all microglia. The procedure of immunohistochemistry was described in detail in our previous reports [18,34,35]. The primary antibodies, including anti-ED-1 and anti-Iba1 (1:100; Abcam, San Francisco, CA, USA) were applied to the samples and incubated at 4 °C overnight. Then, the secondary antibody conjugated with fluorescein isothiocyanate (FITC and rhodamine, 1:100, Jackson ImmunoResearch Laboratories, West Grove, PA, USA) was incubated at room temperature for 1 h. Counterstaining was performed by using DAPI (1:1000, Sigma-Aldrich, St. Louis, MO, USA) to detect the cell nuclei. For comparison, the ED1- and Iba1- positive cells were counted manually at the optic nerve lesion sites in six HPFs (×400 magnification) by using a confocal microscope.

### 2.9. Western Blotting Analysis

The ON samples were collected on day 3 from each group. The protein extracts of the ON were separated by using a 4 to 12% NuPAGE Bis-Tris gel (Invitrogen, Carlsbad, CA, USA). The separated proteins were transferred onto polyvinylidene difluoride membranes. The membranes were blocked by using 5% nonfat milk in Tris-buffered saline/Tween-20 solution containing 20 mM Tris-HCl (pH 7.5), 0.5 M NaCl, and 0.5% Tween-20. Subsequently, the membranes were blotted with either anti-p-AKT (1:1000, antibody 8599S; Cell Signaling Technology Inc., Danvers, MA, USA), anti-nuclear factor erythroid 2–related factor 2 (anti-Nrf2; 1:1000, ab137550; Abcam), anti-sirtuin 3 (anti-SIRT3; 1:1000, ab189820; Abcam), or anti-heme oxygenase 1 (anti-HO-1; 1:10,000, ab68477; Abcam) antibody overnight at 4 °C. The membranes were incubated with a secondary antibody conjugated to horseradish peroxidase against the appropriate host species for one hour at room temperature. The developing reaction was performed by using an enhanced chemiluminescent substrate (Perkin-Elmer Life Science, Boston, MA, USA). Thermofisher’s iBright Analysis software was used for the analysis. Precise protein bands were processed for intensity (background subtracted). The results obtained were normalized against that of GAPDH. The statistical analysis was carried out using Mann–Whitney U test.

### 2.10. Statistical Analysis

Every measurement was conducted in a single-blind fashion in this study. The Mann–Whitney U test was used to evaluate the differences among groups in all experiments. Data are presented as the mean ± standard deviation (SD). All statistical analyses were performed using the SPSS statistical software program (SPSS, Chicago, IL, USA). *p*-values less than 0.05 were considered statistically significant.

## 3. Results

### 3.1. Intravitreal Injection of PEG-G-CSF Does Not Affect Visual Function

**Figure 2:**
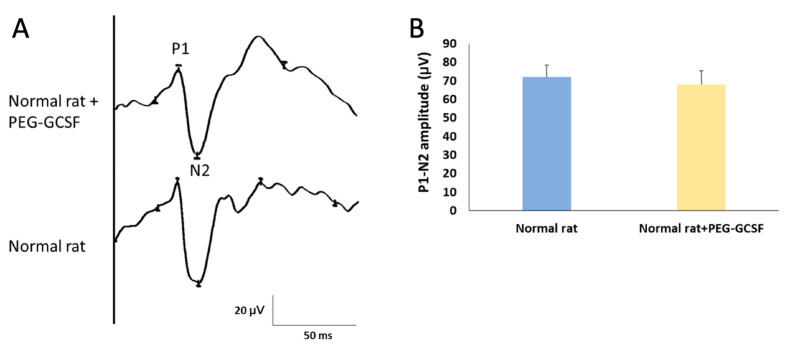
Evaluation of the visual function by using flash visual evoked potentials (fVEPs) in normal rats with or without intravitreal injection (IVI) of pegylated granulocyte colony-stimulating factor (PEG-G-CSF). (**A**) Representative fVEP tracings in the rats treated with or without IVI of PEG-GCSF 14 days later. (**B**) Bar charts demonstrate the P1-N2 amplitude. The values of amplitude are expressed as mean ± standard deviation (SD) in each group (n = 6 in each group). There was no significant difference in the amplitudes of the P1-N2 waves between the rats treated and not treated with IVI of PEG-G-CSF. P1, the first positive peak; N2, the second negative peak.

We first measured the change of fVEP to evaluate visual function two weeks after IVI of PEG-G-CSF. The amplitude of the P1–N2 wavelet can indicate the RGC function in vivo. In this study, we determined the amplitude of the P1–N2 wavelet in the normal Wistar rat, which was 68.61 ± 7.19 μV. The amplitude of the P1–N2 waves in the PEG-G-CSF-treated group was 64.85 ± 9.43 μV (Figure 2). There was no significant difference in the amplitude of the P1–N2 between the PBS-treated group and the PEG-G-CSF-treated group (*p* = 0.72).

### 3.2. Intravitreal Injection of PEG-G-CSF Does Not Induce Leukocytosis

**Figure 3:**
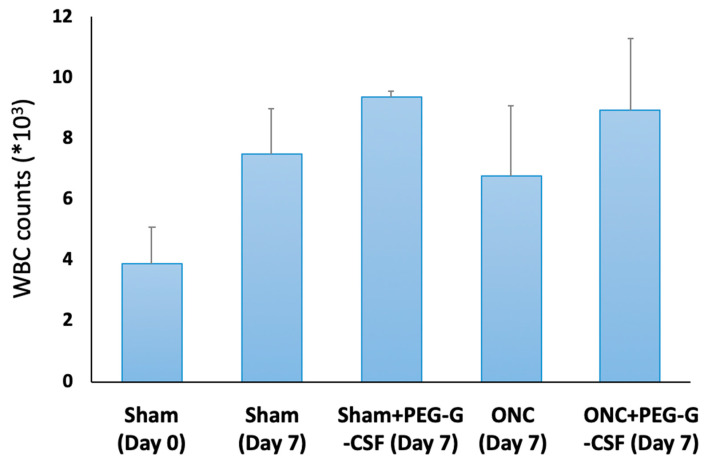
Analysis of white blood cell (WBC) counts in the sham-operated rats with or without intravitreal injection (IVI) of pegylated granulocyte colony-stimulating factor (PEG-G-CSF), and in the optic nerve crushed (ONC) rats with or without IVI of PEG-G-CSF. Though ONC only induced increased WBC counts in peripheral blood, additional IVI of PEG-GCSF did not make a significant difference between the sham-treated group and the ONC group 7 days later.

The peripheral WBC count was checked on day 0 and day 7 after treatment (Figure 3). The number of WBCs in the peripheral blood was 3.9 ± 1.2 × 10^3^/mL in the sham group on day 0, while it was 7.5 ± 1.5 × 10^3^/mL seven days later after sham operation. The WBC counts were slightly elevated in the PEG-GCSF-treated sham group (9.4 ± 0.2 × 10^3^/mL), in the ONC group with no treatment (6.8 ± 2.3 × 10^3^/mL), and the PEG-GCSF-treated ONC group (8.9 ± 2.4 × 10^3^/mL). However, there was no significant difference in peripheral WBC counts between ONC groups with or without IVI of PEG-GCSF (n = 6, *p* = 0.72). IVI of PEG-G-CSF did not induce significant leukocytosis in the injured animals.

### 3.3. Treatment with PEG-G-CSF Protects Visual Function

**Figure 4:**
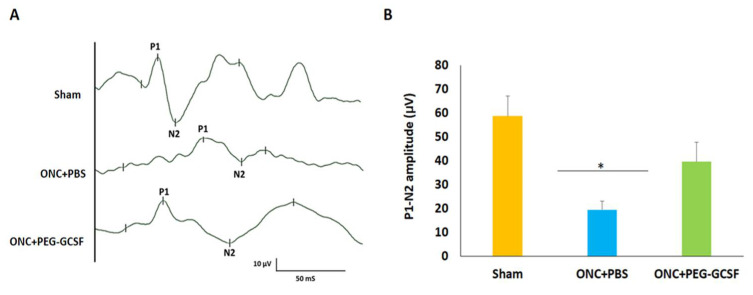
Assessment of visual function by flash visual-evoked potential (fVEP) examination 14 days after optic nerve crush (ONC). (**A**) Representative records of fVEP wavelets in the sham group, the phosphate-buffered saline (PBS)-treated and the pegylated granulocyte colony-stimulating factor (PEG-G-CSF)-treated ONC groups. (**B**) The bar chart showed the measure of the peak to peak amplitudes of P1–N2. There was a significant improvement in the fVEP in the PEG-G-CSF-treated group as compared with that in the PBS-treated group after ONC (* *p* < 0.05, n = 6). P1, the first positive peak; N2, the second negative peak. Data are expressed as mean ± standard deviation (SD).

fVEP was arranged to evaluate the visual function of these experimental rats in the sham group, the PBS-treated, and the PEG-G-CSF-treated ONC groups (Figure 4A). The P1 latency did not exhibit a significant difference between the sham group and the PEG-G-CSF-treated ONC group. The amplitudes of the P1-N2 components in the PEG-G-CSF-treated ONC group were significantly higher than the PBS-treated ONC group (39.6 ± 8.1 vs. 19.5 ± 3.6; *p* < 0.05; Figure 4B).

### 3.4. Treatment with PEG-G-CSF Improves RGCs Survival and Declines Their Apoptosis

**Figure 5:**
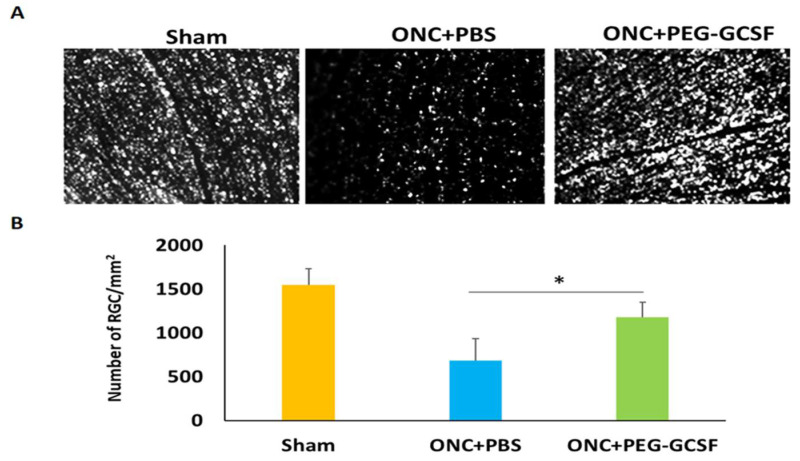
Morphometry and survival of retinal ganglion cells (RGCs) in the sham-operated rats and the optic nerve crushed (ONC) rats treated with intravitreal injection (IVI) of phosphate-buffered saline (PBS), or IVI of pegylated granulocyte colony-stimulating factor (PEG-G-CSF) two weeks after operation. (**A**) Representative images of flat-mounted central retinas and the RGCs labeled by Fluoro-Gold at two weeks post-operation in each group. (**B**) RGC density in the central retina in each group. The number of RGCs in the PEG-G-CSF-treated group was 1.7-fold higher than that in the PBS-treated group. Data are expressed as mean ± standard deviation (SD) for each group (n = 6). (*: *p* < 0.05).

**Figure 6:**
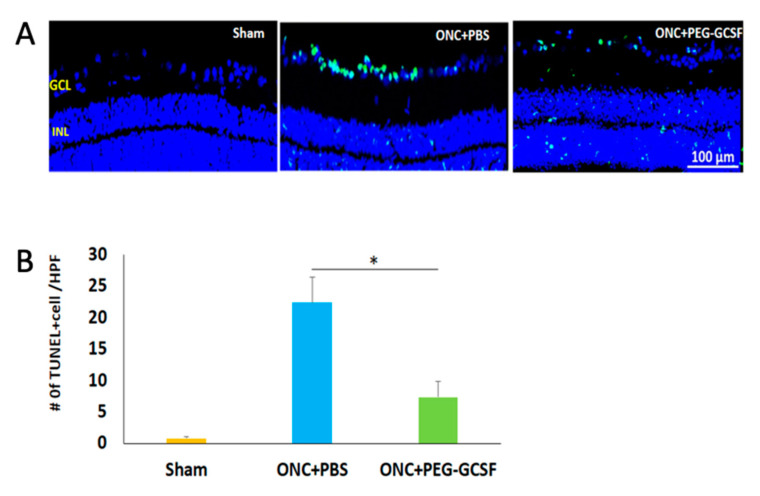
Measurement of retinal ganglion cell (RGC) apoptosis in the RGC layer by in situ Terminal deoxynucleotidyl transferase dUTP nick end labeling (TUNEL) assay at two weeks post-sham-operated and post-optic nerve crushed (ONC) with intravitreal injection (IVI) of phosphate-buffered saline (PBS), or IVI of pegylated granulocyte colony-stimulating factor (PEG-G-CSF). (**A**) Representative images of double-stained apoptotic cells in the retina in each group. The apoptotic cells were stained in green, and the cell nuclei were counterstained with DAPI staining in blue. (**B**) Quantification of apoptotic cells in RGC layer per high-power field (HPF). Treatment with PEG-G-CSF significantly reduced the number of apoptotic RGCs by 3.1-fold when compared with the PBS-treated group. Data are expressed as mean ± standard deviation (SD) for each group (n = 6). (*: *p* < 0.05).

The PEG-G-CSF-treated group preserved a higher density of RGCs in the central retinas than the PBS-treated group (Figure 5A). Two weeks after ONC was conducted, the RGC densities in the central retinas in the sham group, the PBS- and the PEG-G-CSF- treated groups were 1546.4 ± 188.4/mm^2^, 686.4 ± 245.5/mm^2^, and 1181.5 ± 167.5/mm^2^, respectively (Figure 5B). The number of RGCs in the PEG-G-CSF-treated group was 1.7-folds higher than the PBS-treated group (*p* < 0.05). These apoptotic cells in the RGC layers in the sham group, the PBS- and the PEG-G-CSF- treated groups were 0.8 ± 0.3/HPF, 22.4 ± 4.1/HPF, and 7.3 ± 2.6/HPF, respectively (Figure 6A,B). Treatment with PEG-GCSF significantly reduced the number of apoptotic RGCs by 3.1-folds (*p* < 0.05) when compared with treatment with PBS.

### 3.5. Treatment with PEG-G-CSF Inhibits Macrophage Infiltration and Induces Microglia Activation

**Figure 7:**
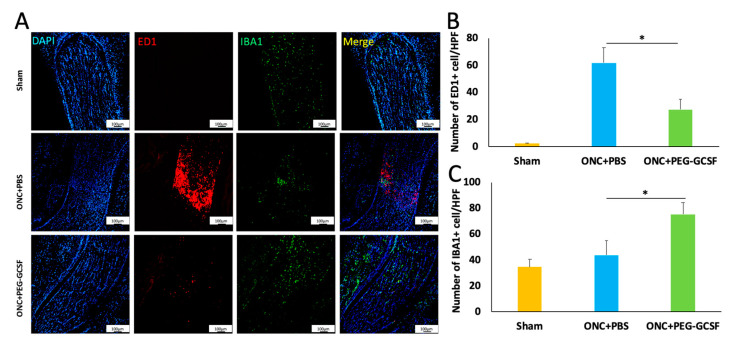
Immunohistochemistry of ED1 and Iba1 in sections of optic nerves (ONs) two weeks post-operation of the sham or the optic nerve crush (ONC). (**A**) Representative images of ED1 and Iba1 staining in longitudinal sections of the ONs. The ED1-positive cells were marked in red color, while the Iba1 were marked in green color. The cell nuclei were labeled in blue color. (**B**) Quantification of ED1-positive cells per high-power field (HPF). The number of ED1-positive cells was significantly lower in the pegylated granulocyte colony-stimulating factor (PEG-G-CSF)-treated group when compared with the phosphate-buffered saline (PBS)-treated group (* *p* < 0.05). (**C**) Quantification of Iba1-positive cells per HPF. The number of Iba1-positive cells was significantly higher in the PEG-G-CSF-treated group when compared with the PBS-treated group (*: *p* < 0.05). Data are expressed as mean ± standard deviation (SD) (n = 6 per group; scale bar = 100 μm).

Iba1 is a cytoplasmic protein expressed in monocyte lineage cells and the brain; it is primarily restricted to microglia in all stages [36,37]. ED1 is known as a useful macrophage marker in rats [38] and is expressed in microglia in the phagocytic stage [39], but not in the resting stage, which may be associated with neuronal damage [40,41]. PEG-G-CSF treatment significantly reduced the number of ED1-positive cells in the ONC model (Figure 7A). The numbers of ED1-positive cells/HPF in the sham, the PBS-, and the PEG-G-CSF- treated groups were 2.3 ± 0.5, 61.7 ± 11.3, and 27.4 ± 7.2, respectively (Figure 7B). Macrophage recruitment decreased by 2.2-folds in the PEG-G-CSF-treated group compared with the PBS-treated group (*p* < 0.05). The optic nerve is composed of retinal ganglion cell axons and glial cells which are critical to its functional integrity and homeostasis through maintenance of functional interactions among glia and the axons [42]. The intrinsic microglia were stained with anti-Iba1 (Figure 7A). Quantification analysis showed 34.4 ± 5.8 Iba1-positive cells/HPF in the sham group, 43.7 ± 11.3 Iba1-positive cells/HPF in the PBS-treated group, and 75.2 ± 9.2 Iba1-positive cells/HPF in the PEG-G-CSF-treated group (Figure 7C). The level of intrinsic microglia activation was increased by 1.7-fold by PEG-G-CSF treatment compared to PBS treatment (*p* < 0.05) (Figure 7C).

### 3.6. PEG-G-CSF Protects Cells from Apoptosis and Decreases ER and Oxidative Stress

**Figure 8:**
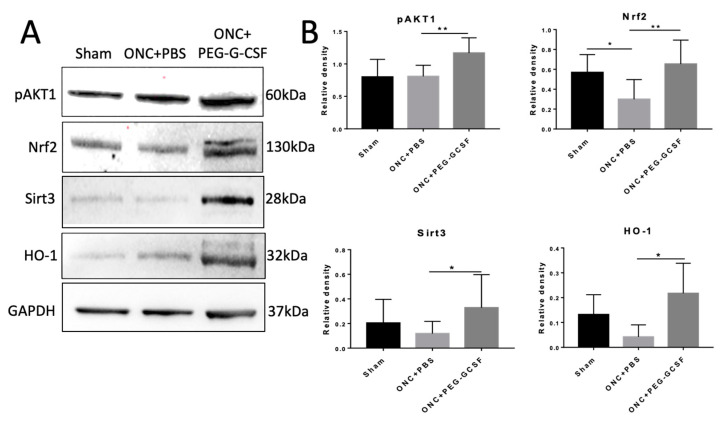
Western blot analyses in the samples of rat optic nerve (ON) post different operations. (**A**) Representative photograph of the immunoblotting of the expression of p-Akt1, Nrf2, Sirt3, and HO-1 in the ON samples. (**B**) Quantification of the protein bands of p-Akt1, Nrf2, Sirt3, and HO-1. Each value was measured by using iBright imaging software and normalized to GAPDH. The expressions of p-Akt1, Nrf2, Sirt3, and HO-1 were significantly higher in the PEG-G-CSF-treated crushed samples than those in the sham group or the PBS-treated crushed samples. Data are expressed as mean ± standard deviation (SD) in each group (n = 6 in each group). *: *p* < 0.05. **: *p* < 0.01.

IVI-PEG-G-CSF up-regulated the expression of p-Akt1 which protects cells from apoptosis by inactivation of apoptotic cascade components. IVI-PEG-GCSF also enhanced the expression of Nrf2, Sirt3, and HO-1, which are important in the modulation of ER stress and oxidative stress. The levels of p-Akt1 and Nrf2 were significantly increased by 1.44- and 2.19-fold, respectively, in the PEG-G-CSF-treated group compared with the PBS-treated group (*p* < 0.01) (Figure 8B upper). The levels of Sirt3 and HO-1 were significantly increased by 2.77- and 5.19-fold, respectively, in the PEG-G-CSF-treated group versus the PBS-treated group (*p* < 0.05) (Figure 8B bottom).

## 4. Discussion

Our previous studies showed that G-CSF has neuroprotective effects on RGCs survival after ONC, in both subcutaneous administration and IVI approach [21]. The subcutaneous injection of G-CSF has a more protective effect on RGCs than that of the IVI [21]. However, the systemic administration of G-CSF may cause leukocytosis and induce unwanted inflammation [43]. In our current study, we proved that IVI of PEG-G-CSF did not interfere with the visual function or induce systemic leukocytosis. IVI of PEG-G-CSF can prevent RGCs from apoptosis and increase their survival rates by reducing the infiltration of macrophages and activation of intrinsic microglia after ONC. IVI of PEG-G-CSF may preserve visual function after ONC via the activation of p-Akt1/Nrf2/HO-1 and the p-Akt1/Nrf2/Sirt3 signaling pathways.

G-CSF is a cytokine and hormone that stimulates the bone marrow to make granulocytes and progenitor cells and recruit them into the peripheral blood [44]. It is approved by the U.S. Food and Drug Administration (FDA) to decrease the incidence of infection in neutropenic patients with non-myeloid malignancies [45]. In a human study, G-CSF reduced total blood leukocyte count as well as retinal WBC density twelve minutes after intravenous injection, but increased total circulating leukocyte counts and retinal white blood cell density eight hours later [43]. We found that the simple IVI of PEG-G-SCF did not affect the visual function by fVEP (Figure 2). IVI of PEG-G-CSF did not induce significant leukocytosis after ONC as an intravenous infusion of G-CSF (Figure 3). By contrast, none of the other hemodynamic parameters, such as retinal red blood cell flux, retinal blood velocities, retinal venous diameter, blood pressure, or pulse rate, were changed by intravenous administration of G-CSF in this human trial [43].

G-CSF was proved to have neuroprotective effects not only in the rat model of anterior ischemic optic neuropathy [17,18,19,20] but also in the optic nerve crush rat model [21,22,23,30]. Our current study showed that IVI of PEG-G-CSF not only preserves the visual function (Figure 4) but also rescues retinal ganglion cells (Figure 5) after ONC which is compatible with our previous studies of subcutaneous injection of G-CSF [21,22,23]. Our TUNEL assay results (Figure 6) and western blot analysis of p-AKT (Figure 8) showed that IVI of PEG-G-CSF has an anti-apoptotic effect on RGCs survival after ONC and this may be related to the p-AKT pathway. This finding is consistent with earlier studies talking about the crucial role of p-AKT which can be activated by G-CSF in neuronal survival after injury [46,47,48]. Nakazawa et al. reported that clamping of ON induced intrinsic activation of p-AKT in the retina within 1 h and reached a maximum at 6 h, then returned to baseline at about 72 h. The inhibition of p-AKT activity significantly lowered the RGC survival rate [49]. Our previous study also showed that subcutaneous administration of G-CSF can activate the p-AKT signaling pathway in the retinas of the injured eyes at 1 and 2 weeks after ONC [23]. These observations suggest that expression of p-AKT after ONC may provide an anti-apoptotic effect and rescue RGCs from apoptosis.

Neuroinflammation is a double-edged sword with both neurotoxic and neuroprotective effects in animal models of traumatic spinal cord injury (SCI) [50,51,52,53,54,55,56]. Extrinsic macrophages and intrinsic microglia belonging to the innate immune system respond quickly to neurotrauma. Microglia are the unique phagocytes as a compensatory mechanism for the immune-privileged status of the central nervous system (CNS) [50,57]. Extrinsic monocytes and macrophages can only enter the CNS in case of disruption to the blood-CNS barrier. Soon after SCI, a localized intensive inflammation is on its way to activate microglia and recruit macrophages from the peripheral blood [58]. The action and distribution of endogenous microglia and infiltrating macrophages have been shown to have things in common but not the same [59,60,61]. Both of these cells play essential roles in the process of neuroinflammation; however, the M2-like macrophages infiltrating after injury may not be enough for efficient debris removal and tissue repair [59]. The different cytokines secreted by microglia and macrophage may have a great impact on the progression of the injured site [62].

Sutherland et al. reported that the higher proportion and numbers of classically activated macrophages and microglia in the rat SCI model may contribute to a more inflammatory microenvironment and lead to further injury [62]. These pro-inflammatory cells are initially essential in SCI to clear damaged cell debris. However, if this process is sustained for too long without progressing to the tissue repair stage dominated by alternatively activated inflammatory cells, it becomes a mess to tissue repair. The presence of these phagocytes at lower levels in the PEG-G-CSF group may be a sign of a less strong pro-inflammatory response, enough to be beneficial but not exceeding the dangerous level. This can be seen in the higher numbers of solely ED1-positive cells in the PBS-treated while the PEG-G-CSF-treated group displayed lower numbers of ED1-positive cells. The significant differences between these 2 groups in the sorely ED1-positive cells may suggest that IVI of PEG-G-CSF helps maintain and repair the highly selective semipermeable blood–brain barrier (BBB) and decrease the infiltration and recruitment of macrophages from peripheral blood as sorely ED1-positive cells [62], which is compatible with our previous findings that early application of G-CSF can stabilize the BBB and reduce inflammation in an rAION model [19]. Therefore, IVI of PEG-G-CSF may contribute to suppressing the flames of neuroinflammation.

Furthermore, an animal study of SCI demonstrated that subcutaneous administration of G-CSF could recruit microglia to the injury site in the first 72 h after spinal cord injury, inhibit the expression of pro-inflammatory factors and promote the expression of neurotrophic factors. G-CSF also increases the expression of markers of M2 macrophage and inhibits the expression of markers of M1 macrophage in vitro, favoring the M2 polarization via the NFκB signal pathway [63]. Many published data show that microglia may run the alternative activation, express M2 markers and make a contribution to neuroprotection [64]. In our previous study, we also found that immediate application of G-CSF can increase the number of activated microglia and trigger alternative activation of M2 microglia/macrophage polarization in the ON in a rAION model [19]. This could be beneficial for ONC rats as there are still neutrophils present to be instrumental but not excessive to have a destructive effect. So, we assume that IVI of PEG-GCSF may reduce early inflammation-related harmful effects and promote an anti-inflammatory response that favors repair by decreasing infiltration of extrinsic macrophage and improving alternative activation of intrinsic microglia.

Cansler and Evanson made a brief commentary on the roles of endoplasmic reticulum (ER) and oxidative stress for the degeneration seen in retinal degeneration, traumatic brain injury (TBI), and TON recently [14]. Neutrophils are the first cells to arrive at the injured area within hours in response to CNS injury [60,61]. They are capable of producing ROS and other neurotoxic factors within the lesion site that can further contribute to lipid peroxidation [65]. Adequate production of oxidative and proteolytic enzymes by infiltrating neutrophils prepares the microenvironment for repair, however, going beyond the limit can cause further injury to the surrounding tissues [66]. Mitochondrial metabolism is relevant to cell redox signaling fine-tuned by protein phosphorylation [67,68], which is the main source of ROS/reactive nitrogen species (RNS) in the cell [69]. Nrf2, a principal key transcription factor, controls the basal and induced expression of an array of antioxidant response element–dependent genes to regulate the physiological and pathophysiological outcomes of oxidant exposure in mitochondrial ROS (mtROS) homeostasis [70]. Kotaro Yamamoto et al. found that mice with ONC showed increased ROS production in the retinal ganglion layer which resulted in reducing survival of RGCs [71]. Our previous study had demonstrated that G-CSF rescues RGCs after ONC is phosphatidylinositol 3-kinase (PI3K)/Akt dependent [22]. Lin Wang et al. reported that the PI3K/Akt pathway plays key roles in regulating Nrf2-antioxidant response element (ARE)-dependent protection against oxidative stress [72]. Joo Choi R et al. also demonstrated that when the PI3K/Akt signaling pathway is activated, phosphorylated Nrf2 can be released from Keap1 and translocated to the nucleus to activate the expression of downstream antioxidant proteins in a lung inflammation model [73]. Silent mating-type information regulation 2 homolog 3 (Sirt3) in the mitochondrial matrix can organize mitochondrial oxidative metabolism, modulate mtROS homeostasis by regulating the mitochondrial complexes in the electron transport chain (ETC), which are considered as the principal sites of mtROS production and the main antioxidant enzymes of mitochondria [74]. Nrf2 can mediate crucial signaling pathways within mitochondria and trigger the activation of Sirt3 to exert a neuroprotective effect under oxidative stress conditions [75]. Antioxidant protein HO-1 has anti-oxidative and anti-inflammatory effects [76,77]. Nrf2 also could increase the transcription of HO-1 against oxidative stress [78]. In our current study, we found the up-regulation of Nrf2, Sirt3, and HO-1 in the western blot analysis of the PEG-G-CSF-treated group (Figure 7). Based on the above findings, it is reasonably assumed that both the p-Akt/Nrf2/Sirt3 and the p-Akt/Nrf2/HO-1 signaling pathways were involved in the salutary effects of PEG-G-CSF on crush-injured ON. This elucidates that the PEG-G-CSF has an anti-oxidant effect in the rat model of TON through IVI administration.

Furthermore, the inflammatory process begins within hours of the insult after CNS damage and lasts for weeks [79]. Recombinant human G-CSF has a short circulating half-life of around 3.5 to 3.8 h [28], which may need frequent injections. A conjugated G-CSF with polyethylene glycol (PEG) helps prolong its half-life up to 42 h in vivo [28]. In this study, we proved that the IVI administration of PEG-G-CSF has the same neuroprotective effect as our previous studies of systemic administration of G-CSF in rAION [17,19,20] and ONC models [21,22,23]. PEG-G-CSF may have a prolonged effect on neuroprotection due to its pegylated formulation and avoid unwanted systemic adverse effects by IVI administration.

However, the main limitation of this study was that we did not evaluate nuclear-fractionated samples to better evaluate the activation of the Nrf2 pathway. The subcellular fractionation approach is an indispensable tool to facilitate the study of specific intracellular events and the characterization of protein functions [80]. Nrf2 is known to be one of the major cytoprotective systems against oxidative stress in the human body [81]. Nrf2 is inactivated by the Kelch-like ECH-associated protein 1 (Keap1) under normal conditions, but detaches from Keap1 and translocates to the nucleus to activate the antioxidant response element (ARE) when encountering oxidative stress, which induces antioxidant response proteins such as HO-1 and mediates cytoprotective effects [82]. Nrf2 acts as an antioxidative transcriptional factor upon accumulation in the nuclei [82]. Therefore, detection of early nuclear translocation of Nrf2 after treatment may help us describe the roles of the Nrf2 pathway in ONC more precisely.

## 5. Conclusions

In summary, we found that IVI of PEG-G-CSF has neuroprotection in a rat model of ONC via anti-inflammation, anti-apoptosis, and anti-oxidation, especially the anti-oxidative stress reaction system involving the p-Akt1/Nrf2/HO-1 and the p-Akt1/Nrf2/Sirt3 axes.

## Data Availability

The data presented in this study are available in the article.
